# Potential Inflammatory Biomarkers for Major Depressive Disorder Related to Suicidal Behaviors: A Systematic Review

**DOI:** 10.3390/ijms241813907

**Published:** 2023-09-09

**Authors:** Ka Young Kim, Ki Young Shin, Keun-A Chang

**Affiliations:** 1 Department of Nursing, College of Nursing, Gachon University, Incheon 21936, Republic of Korea; kykim@gachon.ac.kr; 2 Neuroscience Research Institute, Gachon University, Incheon 21565, Republic of Korea; 3 Bio-MAX Institute, Seoul National University, Seoul 08826, Republic of Korea; 4 Department of Pharmacology, College of Medicine, Gachon University, Incheon 21936, Republic of Korea

**Keywords:** major depressive disorder, suicidal behavior, blood biomarker, inflammation

## Abstract

Major depressive disorder (MDD) is a highly prevalent psychiatric condition affecting an estimated 280 million individuals globally. Despite the occurrence of suicidal behaviors across various psychiatric conditions, MDD is distinctly associated with the highest risk of suicide attempts and death within this population. In this study, we focused on MDD to identify potential inflammatory biomarkers associated with suicidal risk, given the relationship between depressive states and suicidal ideation. Articles published before June 2023 were searched in PubMed, Embase, Web of Science, and the Cochrane Library to identify all relevant studies reporting blood inflammatory biomarkers in patients with MDD with suicide-related behaviors. Of 571 articles, 24 were included in this study. Overall, 43 significant biomarkers associated with MDD and suicide-related behaviors were identified. Our study provided compelling evidence of significant alterations in peripheral inflammatory factors in MDD patients with suicide-related behaviors, demonstrating the potential roles of interleukin (IL)-1β, IL-6, C-reactive protein, C-C motif chemokine ligand 2, and tumor necrosis factor-α as biomarkers. These findings underscore the intricate relationship between the inflammatory processes of these biomarkers and their interactions in MDD with suicidal risk.

## 1. Introduction

Suicide, which transcends geographical, cultural, and socioeconomic boundaries, poses a significant global health challenge [1]. With its rising prominence as a leading cause of mortality worldwide, its devastating social, psychological, and financial impacts are increasingly being recognized [2]. Suicidal incidents frequently occur suddenly in situations of severe distress and are commonly triggered by factors like economic instability, relational discord, chronic pain, or medical disorders. The annual toll exceeds 700,000 fatalities from suicide, and a significantly larger population involved in suicide attempts [1,3]. In the face of such adversity, the endeavor to clinically anticipate the imminent risk of suicide has proven complex. This complexity has redirected academic attention towards unearthing biological correlates for insights into pathogenesis and potential therapeutic and preventive interventions. Because these biological correlations may serve as potential risk indicators [4], the increased suicidal risk in major depressive disorder (MDD) can be diagnosed using certain biomarkers. However, the lack of reliable biological markers for suicidal risk remains a significant issue. This is largely due to an overreliance on self-reported suicidal intentions and the limited predictive power of identified nonbiological risk factors [4]. Nevertheless, our focus in this study was on MDD, aiming to identify potential biomarkers linked to suicidal risk, considering the plausible relationship between depressive states and suicidal ideation [5,6].

MDD is a highly prevalent psychiatric condition affecting an estimated 280 million individuals globally [7]. Characterized by depressive mood; diminished motivation; and a mixed array of cognitive, mental, and physical symptoms, MDD contributes to a significant decline in daily functioning [8]. According to the Diagnostic and Statistical Manual of Mental Disorders-5 (DSM-5), the diagnosis of MDD requires at least five persistent symptoms for a minimum duration of 2 weeks [9]. The disease adversely affects quality of life [10] and contributes to premature morbidity and mortality, primarily through suicidal behavior [10]. Despite the occurrence of suicidal behaviors across various psychiatric conditions, MDD is distinctly associated with the highest risk of suicide attempts and deaths in this population [10,11].

One of the challenges in accurately diagnosing and evaluating MDD is the overlap of symptoms with other neuropsychiatric disorders and the lack of specific biomarkers [12,13]. The lack of definitive biomarkers introduces bias, leading to potential misdiagnosis and affecting disease progression and prognosis. While hereditary factors account for 30–40% of MDD cases, the remaining 60–70% of cases are attributed to nongenetic causes, primarily environmental factors and gene–environment interactions [14]. Identifying significant biomarkers could help refine disease typology, facilitate individualized treatment approaches [15], and ultimately, aid in suicide prevention. Potential biomarkers include indicators of inflammation, neuroendocrine activity, metabolism, growth factors, and alterations in neurotransmitter profiles [16]. However, despite extensive research, there has been limited success in associating definitive biomarkers with MDD using neuroimaging or microRNA analysis [17].

Significant evidence supports an increased prevalence of depressive symptoms in individuals with autoimmune disorders, such as rheumatoid arthritis, psoriasis, diabetes mellitus, chronic inflammatory bowel disease, and autoimmune thyroiditis [18]. Recent findings further suggest a pivotal role of inflammation in the pathogenesis of major psychiatric disorders associated with suicidal behavior, including mood disorders, schizophrenia, and substance use disorders [19]. Diverse immune cells play important roles in the progression of wound healing, wherein they secrete a range of cytokines that trigger both pro-inflammatory cytokines such as interleukin (IL)-1, IL-6, IL-17, and tumor necrosis factor-α (TNF-α); and anti-inflammatory cytokines such as IL-4, IL-10, IL-13, and transforming growth factor-β (TGF-β) [20]. Therefore, we hypothesized that certain inflammatory factors could potentially be utilized as biomarkers associated with the increased risk of suicidal tendencies in MDD. We aimed to explore the association between peripheral inflammatory factors and MDD using them as potential biomarkers. We approached this aim through systematic reviews and meta-analyses and discuss the potential of these inflammatory biomarkers in predicting suicidal risk in MDD.

## 2. Materials and Methods

### 2.1. Literature Search

This systematic review was performed using the Preferred Reporting Items for Systematic Reviews and Meta-Analyses statements [21] and Cochrane Handbook guidelines. This systematic review collected articles published in English from PubMed, Embase, Web of Science, and the Cochrane Library from inception to June 2023. All references were imported into the EndNote X9 reference database. Duplicate articles were deleted using EndNote and manual check. Then, articles were extracted after analyzing the titles, abstracts, and full texts. For this study, we used the following keywords: (“major depress*” OR “unipolar depress*” OR “depress, major” OR “MDD”) AND (inflammat*) AND (blood OR plasma OR serum) AND (suicide*). Two researchers (K.Y. Kim and K.A. Chang) performed the study and collected data independently. Articles were extracted after analyzing the titles, abstracts, and full texts. Any disagreements were discussed by all the authors. 

### 2.2. Selection Criteria

The comprehensive inclusion criteria based on Population, Intervention, Comparison/Comparator, Outcome, Study type (PICOS) were as follows: (1) inclusion of patients with MDD with suicide-related behaviors (suicidal ideation, suicide attempts, or suicidal behavior) compared with healthy controls or patients with MDD; (2) evaluation of inflammatory biomarkers; (3) protein biomarkers found in blood; and (4) randomized controlled trials and epidemiological observational studies, including cross-sectional, case-control, and cohort. The exclusion criteria were as follows: (1) patients with MDD without suicidal risk or those with depressive symptoms not diagnosed with MDD, such as COVID-19 depression; (2) cerebrospinal fluid samples or brain tissue, cellular, and animal studies; (3) gene expression biomarkers, such as microRNA, mRNA, and DNA; and (4) reviews, case reports, conference abstracts, editorials, letters, or commentaries.

### 2.3. Data Extraction and Analysis 

The following data were collected: first author’s name, year of publication, study country, characteristics of groups described in each paper, sample size, sex frequency, mean age by group, diagnostic tool for MDD and suicide-related behavior (including suicidal ideation or suicide attempt), sample preparation, screened inflammatory biomarkers described in each paper, and candidate inflammatory biomarkers described in each paper. The study groups were classified as follows: a control group that did not include MDD and suicide-related behaviors; an MDD group that was diagnosed with MDD, not simple depression, without suicide-related behaviors; an MDD + SI group that included patients with MDD with suicidal ideation; an MDD + SA group that included MDD with suicide attempts; and an MDD group that included other suicidal behaviors.

### 2.4. Statistical Analysis and Quality Assessment 

The standardized mean difference of potential inflammatory biomarkers was analyzed using the Comprehensive Meta-Analysis software version 4 (Biostats Inc. Englewood, NJ, USA). A quality assessment of the articles included in this review was performed using the Critical Appraisal Skills Programme (CASP, Oxford, UK)) randomized controlled trial, CASP case-control, and CASP cohort study checklists [22]. The CASP tool consists of 11 questions in three sections, including “Are the results of the study valid”, “What are the results”, and “Will the results help locally”. 

## 3. Results

### 3.1. Characteristics of the Included Studies 

**Figure 1:**
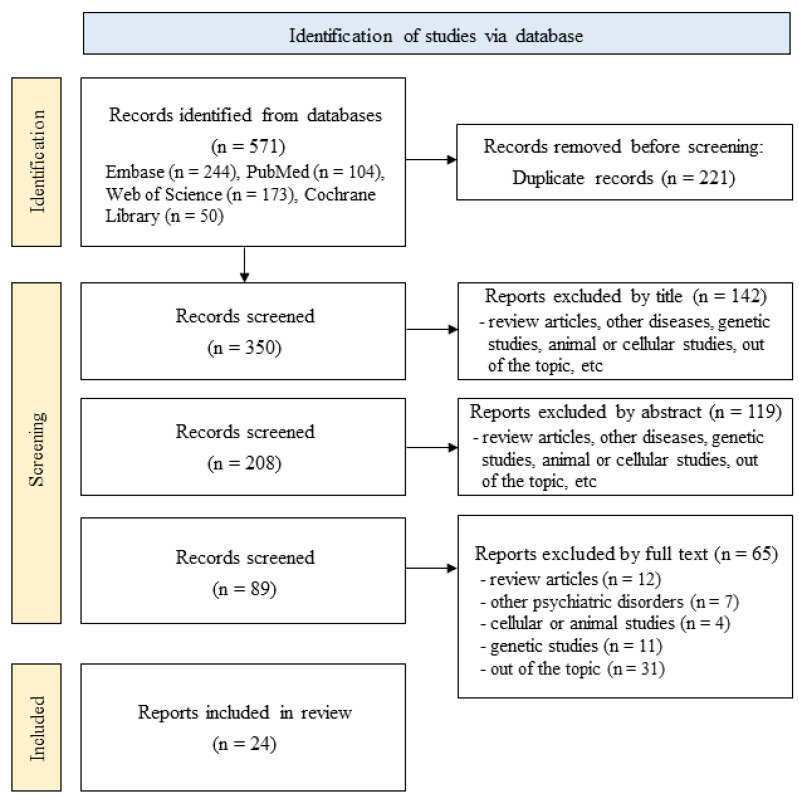
Flow diagram of the literature search.

This systematic review initially identified 571 articles from Embase (244), PubMed (104), Web of Science (173), and Cochrane Library (50) (Figure 1). After eliminating 221 duplicates manually and by using the EndNote Library, 350 articles were manually extracted. Title analysis reduced the number of articles to 208, and abstract screening further reduced it to 89. After deleting review papers, gene expression studies; cellular or animal studies; depressive symptoms not related to MDD; other diseases including bipolar disease, human immunodeficiency virus, and schizophrenia; studies that included only cerebrospinal fluid (CSF) samples; and studies that did not include suicide episodes were excluded. Thus, 24 articles were included in this systematic review after a thorough full-text review. A quality assessment of the articles included was performed using CASP and appropriately evaluated on a scale of 7 to 9 (Table 1).

Table 2 summarizes the studies included in the systematic review. The selected studies were published between 2002 and 2022. The study regions were Israel, China, USA, Sweden, Spain, Brazil, Argentina, Iraq, and Japan. The study groups included healthy controls and patients with MDD, MDD + SI, MDD + SA, or MDD + suicide behavior. Sex and age are presented according to study group. Their ages ranged 18–65 years; more than half of them were in their 30s. The diagnostic tools for MDD were the Beck Depression Inventory, Hamilton Depression Rating Scale, Patient Health Questionnaire, Diagnostic and Statistical Manual for Mental Disorders, and Montgomery–Asberg Depression Rating Scale. Suicide was evaluated using the Beck Scale for Suicide Ideation, Scale for Suicide Ideation, Columbia Suicide Severity Rating Scale-Baseline Assessment, Brief Psychiatric Rating Scale for Children, Suicide Assessment Scale, Center for Disease Control and Prevention, the suicidal module of the Mini International Neuropsychiatric Interview with six questions, and the Diagnostic and Statistical Manual for Mental Disorders. Blood samples included plasma and serum.

### 3.2. Potential Inflammatory Biomarkers in Patients with MDD with Suicide-Related Behaviors

Table 3 shows potential inflammatory biomarkers in patients with MDD with suicide-related behaviors that present in each study. The included studies showed 88 screened biomarkers for inflammatory target analysis. Overall, 43 significant biomarkers associated with MDD with suicide-related behaviors were identified. 

### 3.3. Analyzed Potential Inflammatory Biomarkers

The biomarkers overlapping in two or more articles among all screened biomarkers included in this study were interleukin (IL)-6, tumor necrosis factor (TNF)-α, IL-1β, C-reactive protein (CRP), IL-10, IL-2, C-C motif chemokine ligand (CCL) 2, CCL11, IL-12, IL-1Rα, interferon (IFN)-γ, and IL-4 (Table 4). Among the screened biomarkers, no significant inflammatory biomarkers were identified in the meta-analysis of the two studies with analyzable values. However, the significant biomarkers that were suggested as significant in each article of two or more were IL-6, CRP, TNF-α, IL-1β, and CCL2. Furthermore, potential suicidal ideation biomarkers were IL-6, alpha 1-antitrypsin (AAT), transferrin (TRSF), log CRP, TNF-β, S100B, docosahexaenoic acid (DHA) (%), IL-1β, CCL2, CCL5, IL-12, CCL26, vascular endothelial growth factor (VEGF), IL-17C, CXCL10, and TNF-β; while potential suicide attempts biomarkers were picolinic acid (PIC), PIC: quinolinic acid (QUIN) ratio, log IL-1β, IL-6, DHA (%), vitamin D, mature brain-derived neurotrophic factor (mBDNF), IL-12, zonulin,, intestinal fatty acid binding protein (I-FABP), TNF-α, total cholesterol (TC), low-density lipoprotein (LDL), triglyceride (TG), tenascin-C, a soluble form of the urokinase receptor-type plasminogen activator receptor (suPAR), coagulation factor (F) VII, activated protein C (APC), FV, tissue factor (TF), and tissue factor pathway inhibitor (TFPI). 

## 4. Discussion

Our investigation revealed significant alterations in the peripheral levels of inflammatory factors—specifically IL-1β, IL-6, TNF-α, CRP, and CCL2—in individuals with major depressive disorder (MDD) presenting suicide-related behaviors. These factors are known mediators of inflammation and play a role in the induction, production, and release of proinflammatory signals [47,48]. These cytokines, i.e., IL-1β, IL-6, and TNF-α, though part of distinct superfamilies characterized by structural similarities rather than common genetic origins [48,49], have been linked to neuroinflammation and neurodegeneration [50]. Notably, CRP and CCL2, which are recognized biomarkers of cardiovascular inflammation and neuroinflammation, respectively, have been implicated in the regulation of inflammatory processes [51,52]. Thus, these five factors are associated not only with the inflammatory response seen in patients with MDD suicide-related behaviors, but also with interconnections within the cascade of inflammatory responses. Also, enhanced arrays of inflammatory profiles, combined with the assistance of neuroimaging, have the potential to offer heightened sensitivity and specificity in diagnostic modalities for elucidating the etiology of MDD, as well as furnishing insights for personalized therapeutic strategies [53].

Our findings indicated elevated levels of IL-1β in the peripheral blood of patients with MDD suicide-related behaviors. This cytokine has been associated with impairments in the blood*–*brain barrier and the infiltration of neutrophils into the brain parenchyma [54]. The IL-1 family comprises 11 members, including IL-1α and IL-1β; shares a receptor; and exhibits similar functions, with IL-1β being tightly regulated through macrophage inflammasomes [55,56]. Particularly, IL-1β, TNF-α, and IL-6 can trigger the expression of proinflammatory genes in astrocytes, contributing to neuroinflammation and neurodegeneration [50]. IL-1β, along with IL-6 and TNF, has been linked to sickness behavior and implicated in depressive disorders [57]. Similarly, TNF-α and IL-1β are key cytokines associated with sickness behaviors that overlap with MDD symptoms [58]. Thus, IL-1α and IL-1β levels hold promise as potential biomarkers for MDD with suicide-related behaviors, warranting evaluation of their predictive value, especially when considered alongside other factors.

Furthermore, elevated peripheral levels of IL-6 were observed in patients with MDD suicide-related behaviors. This pleiotropic cytokine is secreted by immune cells and is associated with post-exercise release from the skeletal muscles and potential anti-inflammatory effects during acute exercise [58]. Higher levels of IL-6 and other inflammatory cytokines have been reported in patients with MDD [59], and IL-6 levels decrease following cognitive behavioral therapy (CBT) intervention [60]. IL-6 is strongly associated with suicidal ideation and behavior [61]. Elevated IL-6 levels have been observed in the CSF of patients with MDD and in suicide attempters [62,63,64]. Hence, IL-6 levels hold potential as biomarkers for MDD patients with suicide-related behaviors, warranting investigation of its prognostic impact in conjunction with other factors. 

Our study also highlights elevated peripheral levels of TNF-α in patients with MDD suicide-related behaviors. This crucial proinflammatory cytokine is synthesized by various immune cells and participates in the regulation of inflammatory pathways [65,66]. TNF-α induces the production of CRP, IL-6, IL-1β, and other molecules, and increased proinflammatory cytokine levels have been observed in patients with MDD, especially during mood episodes [67,68]. Elevated plasma TNF-α levels are associated with suicidal tendencies [69,70]. Thus, TNF-α levels could serve as a potential biomarker for MDD in individuals with suicidal behavior, necessitating further exploration of its clinical significance alongside other factors. 

Notably, MDD patients with suicide-related behaviors exhibit higher peripheral levels of CRP, a well-established cardiovascular risk biomarker synthesized under the influence of proinflammatory cytokines [71]. CRP plays a dual role as an inflammatory marker and contributor to the initiation of inflammation [72,73]. Higher CRP levels have been associated with an increased severity of depressive symptoms and risk of future depression [64]. Suicide attempters have reported elevated levels of inflammatory cytokines and CRP [74]. CRP levels hold promise as potential biomarkers for MDD with suicide-related behaviors, and the clinical implications of their prognostic value should be explored further, particularly in conjunction with other factors. 

Finally, our study delved into CCL2 and its distinctive role in MDD with suicide-related behaviors. CCL2, which is implicated in neuroinflammation, neurogenesis, and synaptic plasticity, is produced by various cell types, including monocytes/macrophages [75,76,77,78]. The CCL2*–*CCR2 axis activates microglia and regulates the release of proinflammatory factors [79]. CCL2′s involvement in MDD pathogenesis is supported by human and animal studies [79], albeit with mixed findings related to antidepressant response [80]. CCL2 levels may serve as potential biomarkers for MDD with suicide-related behaviors, necessitating further investigation into their predictive capacity in the context of other relevant factors.

In addition to the five selected inflammatory factors studied, several other potential biomarkers were identified; however, a meta-analysis could not be conducted due to insufficient data. The observed alterations encompassed a wide array of peripheral cytokines, chemokines, and other proteins in MDD patients with suicide-related behaviors, as highlighted in Table 2. Notably, cytokines, such as IL1-Rα, IL-2, IL2-Rα, IL-3, IL-4, IL-5, IL-6, IL-7, IL-8, IL-10, IL-12, IL-12p70, IL-13, IL-15, IL-16, IL-17C, IL-18, IL-27, IL-31, TNF-β, and IFN-γ, exhibited changes similar to those observed in chemokines, such as CCL2, CCL3, CCL4, CCL5, CCL11, CCL17, CCL26, CXCL-1, and CXCL-10. Alterations were also observed in other proteins, such as HDLC, APOA1, SAA1, fibrinogen, GM-CSF, NPY, hypocretin, AAT, α2M, MIF, VEGF, VEGF-C, VEGFR1, FGF basic, ICAM-1, PIGF, FVII, and TF, as well as in factors, such as PIC, QUIN, PIC/QUIN ratio, arachidonic acid, DHA, EPA, TC, HDL, LDL, TG, MPO, TRAP, APC, TLR-1, Tie-2, AOPP, MDA, ERS, FX, and more in MDD patients with suicide-related behaviors. Given these additional data, these factors may emerge as robust biomarkers for MDD with suicide-related behaviors.

We also proposed potential therapeutic approaches for MDD with suicide-related behaviors based on two hypotheses: the inflammation/cytokine hypothesis and the neuroendocrine hypothesis [81]. The inflammation/cytokine theory may provide insights into the susceptibility of individuals with autoimmune conditions and severe infections to depression and the impact of cytokines on mood states [82]. The failure of traditional pharmacological interventions for MDD has driven the exploration of innovative mechanisms, highlighting inflammation and its mediators as integral to the pathophysiology [83]. This has led to the evaluation of non-steroidal anti-inflammatory drugs as potential treatments for MDD [84]. Inflammatory mediators, including cytokines, chemokines, and acute-phase proteins, are elevated in the plasma of patients with MDD [85]. Interestingly, ketamine, an anesthetic, exhibits rapid-acting antidepressant properties owing to its anti-inflammatory effects, which inhibit proinflammatory cytokine production [86,87]. Infliximab, a tumor necrosis factor antagonist, has also been studied for mood disorders [88]. Omega-3 fatty acids, DHA, and EPA, with antioxidant and anti-inflammatory properties, have shown benefits in depression management [89]. Vitamin D modulates proinflammatory cytokines, such as IL-6 and TNF-α, linked with systemic inflammation in depression and suicidal tendencies [90]. Given the emerging role of inflammation in depression [91], several inflammatory biomarkers have the ability to diagnose elevated suicidal risk in MDD, and specific anti-inflammatory agents exhibit the potential to mitigate suicidal behaviors and reduce suicidal ideation.

Another therapeutic approach focuses on restoring immune-neuroendocrine balance by normalizing hypothalamic*–*pituitary*–*adrenal (HPA) axis dysregulation [86]. Dysregulation of the HPA axis and sympathetic nervous system underlies the physiological responses to stress, coordinating the release of corticosteroids and catecholamines to ensure homeostasis [71,92]. A dysregulated HPA axis was observed in patients with suicide-related behaviors, suggesting a dysfunction within this regulatory mechanism [93]. Chronic stress leads to elevated glucocorticoids, stimulating IL-1, IL-6, and TNF-α synthesis [94]. Excessive IL-1β production affects HPA axis activity through a feedback loop, leading to hyperactivity. Dysregulation of the HPA axis is involved in the onset and contribution [95]. Enhanced IL-6 activity in severe depression leads to hypotransferrinemia and hyperactivity of the HPA axis [96]. IL-6 and CRP levels are associated with cognitive impairment, executive function, and attention/working memory [97]. Impaired glucocorticoid receptor sensitivity in MDD reduces the negative feedback, leading to corticotrophin-releasing hormone overproduction and elevated glucocorticoid production [98,99,100]. Thus, targeting HPA axis dysregulation may serve as a promising strategy for developing novel therapeutic targets and enhancing suicide risk prediction.

This study had limitations, including reliance on available data for meta-analysis and not considering the MDD stage and duration. Meta-analysis quality, heterogeneity, and publication bias are challenges; however, our study identified potential biomarkers for MDD with suicide-related behaviors. Additionally, we focused on patients with MDD post-onset, without direct comparisons with other psychiatric disorders. Therefore, further investigation is necessary to compare individuals with MDD with those with various psychiatric disorders to facilitate a comprehensive understanding of the biomarker landscape in patients with MDD with suicide-related behaviors.

In conclusion, our study highlights potential blood-derived biomarkers, including IL-1β, IL-6, TNF-α, CRP, and CCL2, for the diagnosis, prognosis, and progression evaluation of MDD with suicide-related behaviors. These biomarkers enable real-time objective evaluation and aid in diagnosis, outcome prediction, and clinical decisions. The inflammation/cytokine and neuroendocrine hypotheses provide potential therapeutic avenues, and targeting HPA axis dysregulation may hold promise. Our study enhances the understanding of MDD with suicide-related behaviors and offers valuable insights for future research and clinical applications.

## Figures and Tables

**Table 1:** Quality assessment using the CASP checklist.

Questions	1	2	3	4	5	6	7	8	9	10	11	Score out of 11
Amitai et al. [23]	Y	Y	Y	Y	?	?	significant (*p* < 0.05)	can’t tell	Y	?	Y	7
Bai et al. [24]	Y	Y	Y	Y	Y	?	significant (*p* < 0.01)	Precise (95% CI used)	Y	?	Y	9
Bergmans et al. [25]	Y	Y	Y	Y	?	?	significant (*p* < 0.001)	Precise (95% CI used)	Y	?	Y	8
Brundin et al. [26]	Y	Y	Y	Y	?	?	significant (*p* < 0.001)	can’t tell	Y	?	Y	7
Choi et al. [27]	Y	Y	Y	Y	Y	?	significant (*p* < 0.05)	can’t tell	Y	?	Y	8
Coryell et al. [28]	Y	Y	Y	Y	Y	?	significant (*p* < 0.05)	can’t tell	Y	?	Y	8
Falcone et al. [29]	Y	Y	Y	Y	Y	Y	significant (*p* < 0.01)	can’t tell	Y	?	Y	9
Fernández-Sevillano et al. [30]	Y	Y	Y	Y	?	?	significant (*p* < 0.05)	can’t tell	Y	?	Y	7
Ganança et al. [31]	Y	Y	Y	Y	?	?	significant (*p* < 0.001)	can’t tell	Y	?	Y	7
Grassi-Oliveira et al. [32]	Y	Y	Y	Y	?	Y	significant (*p* < 0.001)	can’t tell	Y	?	Y	8
Grudet et al. [33]	Y	Y	Y	Y	?	?	significant (*p* < 0.05)	can’t tell	Y	?	Y	7
Lin et al. [34]	Y	Y	Y	Y	?	Y	significant (*p* < 0.001)	can’t tell	Y	?	Y	8
Lu et al. [35]	Y	Y	Y	Y	?	Y	significant (*p* < 0.05)	can’t tell	Y	?	Y	8
Nowak et al. [36]	Y	Y	Y	Y	?	Y	significant (*p* < 0.05)	can’t tell	Y	?	Y	8
Ohlsson et al. [37]	Y	Y	Y	Y	Y	?	significant (*p* < 0.001)	can’t tell	Y	?	Y	8
Rasheed et al. [38]	Y	Y	Y	Y	Y	?	significant (*p* < 0.05)	can’t tell	Y	?	Y	8
Rui et al. [39]	Y	Y	Y	Y	Y	?	significant (*p* < 0.001)	can’t tell	Y	?	Y	8
Su et al. [40]	Y	Y	Y	Y	Y	?	significant (*p* < 0.05)	can’t tell	Y	?	Y	8
Uchitomi et al. [41]	Y	Y	Y	Y	Y	?	not significant	can’t tell	Y	?	Y	7
Vargas et al. [42]	Y	Y	Y	Y	?	?	significant (*p* < 0.05)	Precise (95% CI used)	Y	?	Y	8
Ventorp et al. [43]	Y	Y	Y	Y	?	?	significant (*p* < 0.001)	can’t tell	Y	?	Y	7
Wiener et al. [44]	Y	Y	Y	Y	?	?	significant (*p* < 0.001)	can’t tell	Y	?	Y	7
Xu et al. [45]	Y	Y	Y	Y	Y	?	significant (*p* < 0.05)	Precise (95% CI used)	Y	?	Y	9
Yang et al. [46]	Y	Y	Y	Y	?	?	significant (*p* < 0.05)	can’t tell	Y	?	Y	7

1. Did the study address a clearly focused issue?; 2. Did the authors use an appropriate method to answer their question?; 3. Were the cases recruited in an acceptable way?; 4. Were the controls selected in an acceptable way?; 5. Was the exposure accurately measured to minimize bias?; 6. (a) Have the authors taken account of the potential confounding factors in the design and/or in their analysis?; 7. How large was the treatment effect?; 8. How precise was the estimate of the treatment effect?; 9. Do you believe the results?; 10. Can the results be applied to the local population?; 11. Do the results of this study fit when other available evidence? Y, yes; N, no; ?, can’t tell; CI, confidence interval.

**Table 2:** Study characteristics of the selected articles.

Author, Year	Country	Group Characteristic (Group = n)	Gender (F/M or Male %)				Age (Mean ± SD or (Median))				Diagnosis Tool		Sample
			HC	MDD	MDD + SI	MDD + SA	HC	MDD	MDD + SI	MDD + SA	MDD	Suicide	
Amitai et al. [23]	Israel	No FLX-associated suicidality = 57 (MDD = 45), FLX-associated suicidality = 35 (MDD = 29)		23 (34%)	12 (40%)			13.9 ± 2.4	13.9 ± 2.5		BDI		Plasma
Bai et al. [24]	China	C = 86, MDD = 20, MDD + SI = 53	52/34	12/8	33/20		37.4 (13.9)	32.3 (14.4)	37.3 (13.2)		HDRS	BSI-CV	Serum
Bergmans et al. [25]	USA	Not depressed = 12,516, Depressed = 867 Not depressed + SI = 199, Depressed + SI = 330	49.7%	33%	44.2%		20 to 65<	20 to 65<	20 to 65<		PHQ-9		Serum
Brundin et al. [26]	Sweden	C = 35, SA = 73(included MDD = 15)	16/19			42/31	40 (19–66)			43 (20–67)	DSM-III		Plasma
Choi et al. [27]	USA	C = 59, MDD = 41, PD = 52	22/37	11/30			38.5 ± 14.6	41.0 ± 16.5			HAM-D	SSI	Serum
Coryell et al. [28]	USA	MDD = 123, MDD + SA = 79		87/36		54/25		38.5 (15.8)		30.7 (12.9)	PHQ-8	CSSRS	Plasma
Falcone et al. [29]	USA	C = 20, Acute psychosis = 40, Mood disorders = 24	50*–*60%		Low risk, 50.0%; High risk, 66.7%				Low risk, 14.5 ± 0.5; High risk, 14.1 ± 0.5		DSM-IV	BPRS-C	Serum
Fernández-Sevillano et al. [30]	Spain	C = 20, MDD = 23, MDD + SI = 33, MDD + SA = 20	14/6	18/5	26/7	13/7	44.6 (9.2)	50.6 (9.9)	44.5 (12.8)	44.7 (8.8)	HDRS		Plasma
Ganança et al. [31]	USA	C = 24, MDD = 38, MDD + SI = 22, MDD + SA = 20	15 (62.5)	6 (16)	12 (55)	10 (50)	33.7 (9.8)	35.1 (9.7)	37.7 (12.0)	32.7 (13.4)	17-HDRS		Plasma/Serum
Grassi-Oliveira et al. [32]	Brazil	C = 16, MDD = 12, MDD + SI = 18		30/0			38.1 (3.9)	37.8 (9.5)	40.2 (8.3)		BDI		Serum
Grudet et al. [33]	USA	C = 14, MDD = 17, MDD + SA = 59	7/7	8/9		34/25	33 (23*–*55)	35 (22*–*54)		38 (18*–*73)	DSM-IV		Serum
Lin et al. [34]	China	C = 96, MDD = 90, MDD + SA = 14	73/23	56/34		14/0	37.5 ± 9.6	38.1 ± 11.4		44.2 ± 12.2	HRSD-24		Serum
Lu et al. [35]	China	C = 22, People who died by suicide (including MDD) = 22	15/7	Suicide (including MDD) 11/11			38.7 ± 16.4	Suicide (including MDD) 38.9 ± 14.0					Plasma
Nowak et al. [36]	Argentina	HC = 20, MDD + SI = 8, MDD + SA = 25			MDD + SI/SA 25/8				MDD + SI/SA 36.4 ± 12.8		DSM-IV		Plasma
Ohlsson et al. [37]	USA	HC = 17, MDD = 13, recent SA = 54	8/9	7/6		30/24	34.4 ± 11.4	34.5 ± 11.5		38.5 ± 14.5	MADRS	SUAS	Plasma
Rasheed et al. [38]	Iraq	C = 30, MDD = 38, MDD + SA = 22	17/13	14/24		6/16	31.1 ± 15.4	30.8 ± 14.1		36.9 ± 10.3	DSM-IV	CDC	Plasma
Rui et al. [39]	China	C = 109, MDD = 86, MDD + SA = 43	56/53	47/39		27/16	32.6 ± 12.8	37.4 ± 14.6		34.8 ± 12.5	HDRS	SSI-Beck	Serum
Su et al. [40]	China	MDD = 118, MDD + suicide risk = 50		95/23	MDD + suicide risk 35/15			39 ± 10.7	MDD + suicide risk 35.9 ± 11.3		HAMD-17	suicidal module of MINI	Serum
Uchitomi et al. [41]	Japan	Cancer patients (MDD = 16, MDD + SI = 8)		7/9	5/3			61.1 ± 11.3	57.6 ± 9.1		DSM-IV/HDRS	DSM-IV	Blood
Vargas et al. [42]	Brazil	Non-smoker: Non-depressed = 123, Depressed = 68 Smoker: Non-depressed = 78, Depressed = 72	Non-smoker 76/47 Smoker 43/35 (including 2 SA)	Non-smoker 54/14 (including 2 SA) Smoker 52/20 (including 22 SA)			18*–*60				DSM-IV/HDRS		Plasma/serum
Ventorp et al. [43]	Sweden	C = 19, MDD = 19, MDD + SA = 54	10/9	10/9		30/24	34.7 ± 10.8	34.0 ± 10.3		38.5 ± 14.5	DSM-IV		Plasma
Wiener et al. [44]	Brazil	HC = 743, MDD = 149 (including suicide risk), BD = 142 (including suicide risk)	354/389	MDD (including suicide risk) 113/36 BD (including suicide risk) 84/58			18*–*35				DSM-IV		Serum
Xu et al. [45]	China	MDD = 26, MDD + SI = 29		19/7	16/13			37.0 ± 2.9	35.5 ± 2.7		DSM-V/HAMD-24		Serum
Yang et al. [46]	China	HC = 12, MDD = 12, MDD + SA = 12	33/16	34/15		31/18	33.1 ± 5.8	36.3 ± 11.2		33.7 ± 12.2	HAM-D		Plasma

C, control; HC, healthy control; MDD, major depressive disorder; SI, suicidal ideation; SA, suicide attempts; FLX, fluoxetine; BDI, Beck Depression Inventory; DSM, Diagnostic and Statistical Manual for Mental Disorders; PHQ-9, Patient Health Questionnaire; HDRS, Hamilton Depression Rating Scale; HAM-D, Hamilton Depression Rating Scale; BSI-CV, Beck Scale for Suicide Ideation—Chinese Version; SSI, Scale for Suicide Ideation; CSSRSBL, Columbia Suicide Severity Rating Scale-Baseline Assessment; BPRS-C: Brief Psychiatric Rating Scale for Children; MADRS: Montgomery*–*Asberg Depression Rating Scale; SUAS: Suicide Assessment Scale; CDC: Center for Disease Control and Prevention; HDRS: Hamilton Depression Rating Scale; SSI-Beck: Beck’s Suicidal Ideation Scale; HAMD-17:17 item Hamilton Depression Rating Scal; MINI: Mini International Neuropsychiatric Interview.

**Table 3:** Potential inflammatory biomarkers in patients with MDD with suicide-related behaviors.

Author, Year	Screened Biomarkers	Candidate Biomarkers
Amitai et al. [23]	Interleukin (IL)-1β, IL-6, tumor necrosis factor (TNF)-α	IL-6
Bai et al. [24]	Alpha 1-antitrypsin (AAT), apolipoprotein A1 (APOA1), C-reactive protein (CRP), high-density lipoprotein cholesterol (HDLC), homocysteine (HCY), transferring (TRSF)	AAT, TRSF
Bergmans et al. [25]	Log C-reactive protein (CRP), log white blood cell count, standardized diet inflammatory index score	log CRP
Brundin et al. [26]	Picolinic acid (PIC), quinolinic acid (QUIN), PIC:QUIN ratio	PIC, PIC:QUIN ratio
Choi et al. [27]	CRP, IL-10, interferon (IFN)-γ, TNF-α	TNF-α
Coryell et al. [28]	CRP, IL-6, IL-1β, IL-1ra, TNF-α	log IL-1β
Falcone et al. [29]	S100B	S100B
Fernández-Sevillano et al. [30]	IL-2, IL-2R, IL-4, IL-6, TNF-α	IL-6
Ganança et al. [31]	Docosahexaenoic acid (DHA; %), eicosapentaenoic acid (EPA; %), IL-6, IL-1β, plasma phospholipid levels of arachidonic acid (AA; %), TNF-α	DHA (%), IL-1β
Grassi-Oliveira et al. [32]	C-C motif chemokine ligand (CCL) 2, CCL5, CCL11	CCL2, CCL5
Grudet et al. [33]	IL-1β, IL-6, TNF-a, vitamin D	Vitamin D
Lin et al. [34]	Mature brain-derived neurotrophic factor (mBDNF)	mBDNF
Lu et al. [35]	Neuropeptide Y (NPY), IL-1β, hypocretin	NPY, IL-1β
Nowak et al. [36]	IL-12, IL-6	IL-12, IL-6
Ohlsson et al. [37]	IL-6, intestinal fatty acid binding protein (I-FABP), soluble CD14, zonulin	I-FABP, IL-6, Zonulin,
Rasheed et al. [38]	IL-1β, high-density lipoprotein (HDL), low-density lipoprotein (LDL), TNF-α, total cholesterol (TC), triglyceride (TG)	LDL, TC, TG, TNF-α
Rui et al. [39]	Tenascin-C	Tenascin-C
Su et al. [40]	Alpha-2-macroglobulin (α2M), CCL-2, chemokine (C-X-C motif) ligand 1 (CXCL-1), CRP, IL-1β, IL1-Rα, IL2-Rα, IL-6, IL-18, macrophage migration inhibitory factor (MIF), myeloperoxidase (MPO), TNF-α, toll like receptor 1 (TLR-1)	CCL-2, CXCL-1, IL-1β, IL2-Rα, TLR-1
Uchitomi et al. [41]	Platelet Ca2+	
Vargas et al. [42]	Advanced oxidation protein products (AOPP), CRP, erythrocytes sedimentation rate (ESR), fibrinogen, lipid hydroperoxides, malondialdehyde (MDA), nitric oxide metabolites (NOx), total reactive antioxidant potential (TRAP)	AOPP, CRP, ESR, Fibrinogen, NOx, TRAP
Ventorp et al. [43]	CRP, soluble form of the urokinase receptor-Type plasminogen activator receptor (suPAR)	suPAR
Wiener et al. [44]	IL-6, IL-10	IL-6, IL-10
Xu et al. [45]	IL-1α, IL-1β, IL-1RA, IL-2, IL-3, IL-4, IL-5, IL-6, IL-7, IL-8, IL-10, IL-12, IL-12p70, IL-13, IL-15, IL-16, IL-17C, IL-27, IL-31, CCL3, CCL4, CCL11, CCL17, CCL26, CXCL10, fibroblast growth factor (FGF) basic (FGF2/bFGF), granulocyte-macrophage colony-stimulating factor (GM-CSF), IFN-γ, intercellular cell adhesion molecular (ICAM)-1, placenta growth factor (PIGF), thymic stromal lymphopoietin (TSLP), Tie-2, TNF-α, TNF-β, vascular endothelial growth factor (VEGF), VEGF-C, VEGFR1	CCL26, CXCL10, IL-17C, TNF-β, VEGF
Yang et al. [46]	Activated protein C (APC), coagulation factor (F) VII, F V, tissue factor (TF), tissue factor pathway inhibitor (TFPI), prothrombin, prothrombin fragment (F)	APC, FV, F VII, TF, TFPI

**Table 4:** Analysis of potential inflammatory biomarkers.

Characteristics of Analysis	Potential Inflammatory Biomarkers
Markers overlapping in two or more articles among all screened variables included in this study	CCL2, CCL11, CRP, IFN-γ, IL-1β, IL-1Rα, IL-2, IL-4, IL-6, IL-10, IL-12, TNF-α
Significant markers in two or more articles	CCL2, CRP, IL-1β, IL-6, TNF-α
Potential SI markers	AAT, CCL2, CCL5, CCL26, CXCL10, DHA (%), IL-1β, IL-6, IL-12, IL-17C, log CRP, TRSF, TNF-α, S100B, TNF-β, VEGF
Potential SA markers	APC, DHA (%), FV, FVII, I-FABP, IL-6, IL-12, LDL, log IL-1β, mBDNF, PIC, PIC:QUIN ratio, suPAR, TC, Tenascin-C, TF, TFPI, TG, TNF-α, vitamin D, zonulin

## Data Availability

The data supporting the findings of this study are available from the corresponding author upon reasonable request.
